# Analysis of renin-angiotensin aldosterone system gene polymorphisms in malaysian essential hypertensive and type 2 diabetic subjects

**DOI:** 10.1186/1475-2840-8-11

**Published:** 2009-02-25

**Authors:** Vasudevan Ramachandran, Patimah Ismail, Johnson Stanslas, Norashikin Shamsudin

**Affiliations:** 1 Genetic Research Group, Department of Biomedical Science, Faculty of Medicine and Health Sciences, Universiti Putra Malaysia, Selangor, Malaysia; 2 Department of Medicine, Faculty of Medicine and Health Sciences, Universiti Putra Malaysia, Selangor, Malaysia

## Abstract

**Background:**

The renin-angiotensin aldosterone system (RAAS) plays an important role in regulating the blood pressure and the genetic polymorphisms of RAAS genes has been extensively studied in relation to the cardiovascular diseases in various populations with conflicting results. The aim of this study was to determine the association of five genetic polymorphisms (A6G and A20C of angiotensinogen (AGT), *MboI *of renin, Gly460Trp of aldosterone synthase and Lys173Arg of adducin) of RAAS genes in Malaysian essential hypertensive and type 2 diabetic subjects.

**Methods:**

RAAS gene polymorphisms were determined using mutagenically separated PCR and PCR-RFLP method in a total of 270 subjects consisting of 70 hypertensive subjects without type 2 diabetes mellitus (T2DM), 60 T2DM, 65 hypertensive subjects with T2DM and 75 control subjects.

**Results:**

There was significant difference found in age, body mass index, systolic/diastolic blood pressure, fasting plasma glucose and high density lipoprotein cholesterol levels between the hypertensive subjects with or without T2DM and control subjects. No statistically significant differences between groups were found in the allele frequency and genotype distribution for A20C variant of AGT gene, *MboI *of renin, Gly460Trp of aldosterone and Lys173Arg of adducin (p > 0.05). However, the results for A6G of AGT gene revealed significant differences in allele and genotype frequencies in essential hypertension with or without T2DM (p < 0.001).

**Conclusion:**

Among the five polymorphisms of RAAS genes only A6G variant of AGT gene was significantly associated in Malaysian essential hypertensive and type 2 diabetic subjects. Therefore, A6G polymorphism of the AGT gene could be a potential genetic marker for increased susceptibility to essential hypertension with or without T2DMin Malaysian subjects.

## Background

It is well known that the renin angiotensin-aldosterone system (RAAS) as a circulating or hormonal system that regulates blood pressure, electrolyte and fluid homeostasis [1]. The development of hypertension in diabetes is also due to the effects of RAAS activation [2]. On the basis of biochemical or physiological functions, genetic variants of angiotensin converting enzyme, angiotensinogen (AGT), angiotensin type 1 receptor, aldosterone synthase (CYP11B2), adducin, renin of RAAS have been intensively studied in various populations with conflicting results in relation to hypertension [3], diabetic complications [4], coronary heart disease [5] and renal disease [6].

After an initial report [7], several studies analyzed the association of AGT variants in relation to hypertension [8] and diabetes [9] with conflicting results. Among the variants of AGT gene, A-20C variant (adenine-to-cytosine transition at nucleotide -20) of the 5' upstream core promoter region affects the transcription activity of AGT mRNA and thereby alters the plasma AGT level and possibly involved in the development of EHT [10]. Another variant A for G substitution at position -6 in the core promoter of the gene has been significantly associated with elevated plasma AGT levels, AGT gene transcription and EHT [11].

Renin plays a crucial role in the regulation of blood pressure, and the renin gene (*REN*) is considered as a good candidate quantitative trait locus involved in the etiology of essential hypertension. The variations in the amount of linkage disequilibrium have been observed at the human *REN *gene locus indicating the importance of investigating several REN gene polymorphisms. *REN MboI *two-allele polymorphism of *REN *gene shows a positive association with susceptibility to EHT [12,13] and also negative associations [14].

Aldosterone plays an important role in controlling the sodium balance, intravascular volume and in regulating the blood pressure [15]. Aldosterone is synthesized from deoxycorticosterone in the zona golmerulosa of adrenal cortex by a mitochondrial cytochrome P450 enzyme, CYP11B2 [16]. Mutations in CYP11B2 can lead to important changes to arterial pressure and could be responsible for hypertension. The haplotypes in CYP11B2 is strongly associated with hypertension or plasma aldosterone concentrations [17]. An amino acid polymorphism showing Lys173 rather than Arg173 in *CYP11B2 *gene has been associated with low-renin hypertension in Japanese populations [18].

Adducin is a heteromeric cytoskeleton protein composed of α and β-subunits was the rate-limiting factor in RAAS,. Variation in the α-adducin (ADD1) protein may affect ion transport through modification of actin cytoskeleton assembly and modulation of sodium pump activity [19] and it has been suggested that defects in transport mechanisms may be the cause of hypertension. Several studies have suggested that the association or linkage of G- to-T substitution polymorphism of amino acid residue 460 at nucleotide position 614 of exon 10 of the α-Adducin gene in EHT with controversial results in different populations [20].

Hypertension is an extremely common co-morbidity of diabetes and affects 70% of patients with diabetes and is approximately twice as common in persons with diabetes as in those without diabetes [21]. Several epidemiologic studies show that the evidence for co-existence of hypertension and diabetes is due to common genetic and environmental factors such as diet, physical activity and age interact with the genetic predisposition, leading to the development of cardiovascular disease [22]. The pathogenesis of hypertension in diabetes is complex and involves several unified factors that collectively increase the susceptibility to develop hypertension. There is a strong association between up-regulation of RAAS, hypertension and diabetes [2]. A short-term moderate hyperglycemia has been linked to an increase in plasma renin activity, mean arterial pressure and renal vascular resistance with activation of circulating and the local RAAS [23]. RAAS genes are responsible for excess Angioyensin II production or availability are possible candidates for development of diabetic complications and the RAAS gene polymorphisms has been extensively studied in various populations in relation to diabetic complications [24].

The screening of candidate genes for nucleotide variants that are associated with EHT and T2DM is a core component of much diabetes and hypertension genetics research. Several studies of the genetics of EHT and T2DM have been analysed to select the candidate genes because of known or presumed biological or physiological functions. The choice of candidates is inevitably limited by our incomplete understanding of the regulation of the processes and the pathophysiology of EHT and T2DM. Moreover there was no gene consistently associated with or linked to EHT and T2DM [25,4].

The present study was undertaken to determine the possible association of two genetic variants of AGT gene and one polymorphism from each of these candidate genes; *REN*, CYP11B2 and ADD1 in Malaysian EHT with or without T2DM and T2DM subjects.

## Methods

### Study subjects

A total of 195 patients were recruited from Universiti Putra Malaysia (UPM) Physician Clinic, Hospital Kuala Lumpur. Unrelated healthy individuals were collected randomly (no age-sex matched controls) from UPM staff members and volunteers. The subjects were divided into four main groups: EHT without T2DM (group 1, No-70), T2DM (group 2, No-60) EHT with T2DM (group 3, No-65) and control subjects as group 4 (No-75). A questionnaire in both Malay and English languages were obtained to asses the socio-demographic factors. Informed consent was obtained from all the subjects who have participated in this study. The protocol of this study was approved by the Ethical Committee of the Faculty of Medical and Health Sciences, Universiti Putra Malaysia (UPM/FPSK/PADS/T-TAD/T7-MJKEtikaPPer/F01).

### Physical measurements

Individual weight and heights were obtained to calculate body mass index (BMI) using the formula, weight (kg)/[height (m2)]. Hypertension was defined as ≥ 140 mmHg of systolic blood pressure (SBP) and ≥ 90 mmHg of diastolic blood pressure (DBP) or who were receiving anti-hypertensive therapy. The blood pressure was measured on the right arm of the subjects using an automated blood pressure monitor (Omron, Japan) by seated and rested for 5 minutes. The fasting blood glucose levels were obtained from the medical records of all the known diabetic subjects. For control subjects, fasting blood glucose level was measured using MediSense Precision-G blood glucose monitor.

### DNA preparation

Four-five milliliters of blood samples were collected from the peripheral venous blood into an EDTA tube (Becton Dickinson, NJ) by a qualified phlebotomist. Genomic DNA extraction was carried out using the DNA isolation kit (BioBasic.Inc, Canada). The purity of extracted DNA was quantified using Eppendorf UVette^® ^in Biophotometer (Eppendorf, Hamburg, Germany).

### Biochemical analysis

Before the DNA extraction, serum was separated from the blood by centrifuge at 4000 rpm and stored at -20°C until further analysis. Serum levels of high-density lipoprotein-cholesterol (HDL-C), total cholesterol (TC) and triglycerides (TG) were measured enzymatically on a Hitachi-912 Autoanalyser (Mannheim, Germany) with kits supplied by Roche Diagnostics (Mannheim, Germany). Low density lipoprotein cholesterol (LDL-C) was calculated using Friedewald formula [26].

### Determination of RAAS gene polymorphisms

Detection of A6G polymorphism of AGT gene and Gly460Trp polymorphism of ADD1 was performed by mutagenically separated PCR (MS-PCR) technique [27] with both normal and mutant alleles were added together in one reaction tube. The other polymorphisms (A20C, *MboI *and Lys173Arg) were assayed by restriction analysis of the PCR product with the respective restriction enzymes.

The A6G polymorphism of AGT gene was amplified in a 25-μL reaction mixture contains the template DNA, 10 pmol/L of each three primers, 0.4 mmol/L each dNTP, 2 mmol/L MgCl_2_, 1× Taq buffer and 1 unit of NEB *Taq *DNA polymerase (New England Biolabs, Beverly, MA, USA). The temperature for initial denaturation step was 94°C for 5 min, followed by 35 number of cycles, each of 94°C for 45 seconds (s), 62°C for 45 s, and 72°C for 45 s, and by a final extension at 72°C for 7 min. The amplification reaction yields a 187-bp and a 207-bp product for the A-6 and G-6 alleles respectively [28].

PCR amplification was performed for Gly460Trp polymorphism of ADD1 gene in a 25-μL reaction mixture contained 2.4 pmol/L of forward primer (FP)-1, 4.8 pmol/L of FP-2 and 6 pmol/L of reverse primer (RP), 0.4 mmol/L each dNTP, 2 mmol/L MgCl_2_, 1× Taq buffer and 1 unit of NEB *Taq *DNA polymerase (New England Biolabs, Beverly, MA, USA) and the template DNA. The initial denaturation was set up for 3 minutes at 95°C followed by 35 cycles of denaturation for 20 s at 94°C, annealing for 30 s at 60°C, and extension for 30 s at 72°C [29-31]. The size of the PCR products was 220 bp and 234 bp for the 460Gly and 460Trp alleles, respectively.

To determine the genotypes of *MboI *of *REN*, Gly460Trp of CYP11B2 and Lys173Arg of ADD1 genes, genomic DNA was amplified first by the respective primers (Table 1) using polymerase chain reaction (PCR) technnique. The PCR amplification for all the respective polymorphisms was performed in total volume of 25-μL reaction mixture consisting of 10 pmol of each primer, 0.3–0.4 mmol/L each dNTP, 1.5–3.0 mmol/L MgCl_2_, 1× *Taq *buffer and 1 unit of NEB *Taq *DNA polymerase. The PCR products of the respective primers was digested with 2–4 units of the respective restriction enzymes (New England Biolabs, Beverly, MA, USA) and incubated at 37°C for 3–5 hours with the respective NEB buffers in a final volume of 20 μL reaction mixture. The heat inactivation was done on the basis of manufacture's protocol for the respective restriction enzymes. A negative control containing no genomic DNA and a positive control of known genotype were always included in the set of reactions. All the PCR cycling conditions were carried out on iCycler machine (BioRad Laboratories, Hercules, California, USA). The amplified PCR products for A6G and Gly460Trp polymorphisms were separated on a 3% Metaphor agarose gel (Cambrex, East Rutherford, NJ, USA), while other polymorphisms were separated at 2–4% of agarose gel (Bioline, London, UK) and performed in Origins electrophoresis tank (Elchrom Scientific AG, Switzerland). The agarose gel was stained in ethidium bromide and visualized using Alpha Imager (Alpha Innotech, San Leandro, CA). Table 1 shows the primers used for RFLP method, PCR cycling conditions, restriction endonucleases and the digested restricted fragment size products. Identical results were obtained when genotyping was performed for 10% of the samples on two separate occasions.

**Table 1:** Oligonucleotides for amplification and screening for three polymorphisms using PCR-RFLP method

Gene polymorphism	Forward primer (FP) Reverse primer (RP)	PCR cycling conditions	Restriction endonuclease	PCR product (bp)	Restriction fragment size (bp)
A20C	FP-5'-AGA GGT CCC AGC GTG AGT GTC-3' RP-5'-AGC CCA CAG CTC AGT TAC ATC-3'	95°C- 3 m	*EcoO109I *[10]	265	A-265 C-205,137
		94°C- 1 m			
		64°C -1 m			
		72°C -1 m			
		72°C-5 m			
		X 30			

*MboI*	FP-5'-GAG GTT CGA GTC GGC CCC CT-3' RP-5'-TGC CCA AAC ATG GCC ACA CAT-3'	94°C- 5 m	*MboI *[30]	250	*MboI *(-) -250
		94°C- 30 s			*MboI *(+) -171, 79
		68°C- 30 s			
		72°C- 30 s			
		72°C-5 m			
		X 30			

Lys173Arg	FP- 5'-AGG CAG CTT CTA CCA GGG CCC CAG TCA CT-3' RP- 5'-CCC CTC CCC TGC AAA TCT CAT CCC TTA-3'	94°C- 3 m	*Bsu36I *[31]	1286	L-1286, A- 1037, 249
		94°C -45 s			
		61°C -45 s			
		72°C -1 m			
		72°C-10 m			
		X 35			

PCR cycling conditions are represented as temperature and time [minute (m), seconds (s] of initial denaturation, denaturation, annealing, extension and final extension X number of cycles.

### Statistical analysis

All the statistical analysis was carried out by using SPSS (Chicago, IL) software version 14.0 for Microsoft Windows. Continuous variables were compared between the groups by using two-tailed student's *t *test. Allelic frequencies were calculated by gene-counting method and the genotype distribution with Hardy-Weinberg expectations by a chi-squared test. Odds ratios (OR) with 95% confidence intervals (CI) were estimated for the effects of high risk alleles. A level of *P *< 0.05 was considered statistically significant.

## Results and discussion

In detecting the association between the genetic polymorphisms and trait using association studies remains controversial. However, it is an efficient method to evaluate the associations of allele frequency or genotype frequencies of candidate genes with diseases to understand the genetic etiology of complex human traits [32]. Taking this into an account, in this present study we determined the possible association of genetic polymorphisms of RAAS genes with EHT and T2DM in Malaysian subjects. To our knowledge, few papers have been published in relation to RAAS gene polymorphisms in Malaysian subjects. Probably this is the first comprehensive reports on RAAS pathway gene polymorphisms in relation to EHT and T2DM in Malaysian subjects.

### Baseline characteristics

A total of 270 subjects were recruited in this study. Among the subjects, 70 (25.93%) were hypertensive, 60 (22.22%) T2DM, 65 (24.07%) were hypertensive with T2DM and 75 (27.78%) unrelated healthy individuals. The majority of the subjects in this study were males [163 (60.37%)] compared to female subjects [107 (39.63%)]. Table 2 summarizes the clinical parameters of all the subjects. According to American Diabetes Association, age plays a significant role in the risk of developing T2DM and it is most often seen in older adults. In this study, the median age in the patient group was 57.21 years and the range was 31–84 years as compared to control group (median 45.37 years, range 30–74). Since the control group is younger than the patient group, there was significant differences found in age in all the three groups as compared with control group (p < 0.05). This is similar to a study done in Malaysian hypertensive subjects [33] which shows that, the hypertensive subjects ranged from 30 to 78 years old, with a mean age of 54.7 years, while the normotensives ranged from 25 to 78 years old, with a mean 49.3 years, but, in contrast to the findings in Malays [34].

**Table 2:** Baseline characteristics of the subjects

Parameters	Group 1 (n-70)	Group 2 (n-60)	Group 3 (n-65)	Group 4 (n-75)
Gender (male/female)	51/19*	33/27	40/25	39/36
Age (Years)	57.70 ± 11.02*	57.70 ± 10.24*	57.61 ± 9.76*	45.37 ± 10.69
BMI (kg/m2)	25.63 ± 4.15*	27.89 ± 3.93*	26.71 ± 3.59*	23.91 ± 4.01
SBP (mm Hg)	159.44 ± 17.10*	125.93 ± 9.05	160.26 ± 19.01*	125.98 ± 8.81
DBP (mm Hg)	96.40 ± 6.52*	74.97 ± 7.34	93.60 ± 3.77*	77.83 ± 7.09
FPG (mmol/L)	5.24 ± 0.54*	11.85 ± 4.81*	11.68 ± 4.71*	4.60 ± 0.91
HDL-C (mmol/L)	0.86 ± 0.31*	0.73 ± 0.22*	0.78 ± 0.29*	1.11 ± 0.40
LDL-C (mmol/L)	3.42 ± 1.09	4.04 ± 1.47*	3.76 ± 1.19	3.36 ± 1.16
TG (mmol/L))	1.97 ± 0.99	2.09 ± 1.01*	1.95 ± 0.87	1.64 ± 0.98
TC (mmol/L	4.80 ± 1.10	5.14 ± 1.31	5.05 ± 1.26	5.12 ± 1.38
Na (mEq/L)	144.17 ± 12.78*	133.01 ± 9.60*	137.12 ± 10.11*	114.73 ± 18.38
K (mEq/L)	4.94 ± 1.15	4.90 ± 0.82	5.24 ± 0.92	5.13 ± 0.89

Data presented as mean ± SD, Group 1- EHT, Group 2 -T2DM, Group 3-EHT+T2DM and Group 4-Control subjects,* P value < 0.05 vs. Group 4

The subjects were stratified according to gender in hypertensive subjects [51 (72.86%) males and 19 (27.14%) females], T2DM [33 (55%) males and 27 (45%) females], T2DM with EHT [40 (61.54%) males and 25 (38.46%) females] and unrelated healthy individuals as controls [39 (52.00%) males and 36 (48.00%) females]. There was a significant difference found in hypertensive subjects as compared to normotensive subjects for gender which was not similar to the prevalence of hypertension found in Malaysian population [35]. The study shows [35] that the prevalence of hypertension in males and females was not significantly different above the age of 40 years, however, aged 15–39 years, hypertension was significantly more prevalent in males than females (P < 0.05) in Malaysian population. Significant differences (p < 0.05) was observed in age, BMI, DBP, FPG, HDL-C and Na in the patient groups as compared to the control subjects. In hypertensive subjects with or without T2DM, SBP, DBP, BMI, FPG and Na was in higher levels while HDL-C was lower in levels as compared to control subjects and significantly differed than those in controls. The variables of SBP and DBP at the time of participation were significantly higher in hypertensive groups than control group (p < 0.001). The results, support the previous hypothesis that the higher the blood pressure, the greater the risk of heart attack, heart failure, stroke and kidney disease [36]. The presence of additional risk factors such as BMI and high cholesterol levels increases the CVD risk from hypertension [36]. However, other risk factors such as TC, high LDL-C and low potassium were not significantly different with control subjects. Among the patient groups, obesity was clearly observed in T2DM (27.89 ± 3.93) as compared to other subjects. However, in hypertensive subjects with or without T2DM were under pre-obesity category 25.63 ± 4.15 and 26.71 ± 3.59 respectively as compared to overweight control subjects (23.91 ± 4.01). The BMI for the subjects in the patient groups were significantly different from control subjects (p < 0.05) for BMI. Elevated serum triglycerides strongly indicate the presence of metabolic syndrome in patients with T2DM [37]. Only in type 2 diabetic patients, the concentration of LDL-C was significantly different from control subjects and the type 2 diabetic subjects are at borderline high category (>3.36 mmol/l) according to NCEP ATPIII [38]. There was no significant difference found in LDL in hypertensive subjects with or without diabetes as compared to control group (p > 0.05). Triglycerides was significantly differed only in diabetic subjects (p < 0.05) but not in other groups. Total cholesterol was not significantly differed between the patient groups and the control subjects as they found under desirable category according to NCEP ATPIII. The aggregation of risk factors found in our study was another determinant of the early occurrence of the disease. An elevation in TG, and a reduction in HDL-C favored the hypothesis that metabolic syndrome participates in the development of early CAD [39].

### Genotyping results

**Figure 1:**
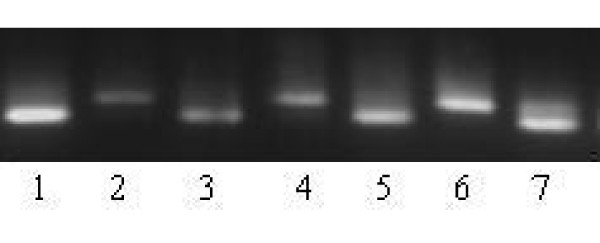
**Agarose gel electrophoresis showing the genotypes of RAAS gene polymorphisms**. Llane 1, 3 and 5 represents homozygous AA genotypes, lane 2, 4 and 6 represents homozygous GG genotypes and lane 7 shows the heterozygous AG genotypes of A6G polymorphism of AGT gene.

**Figure 2:**
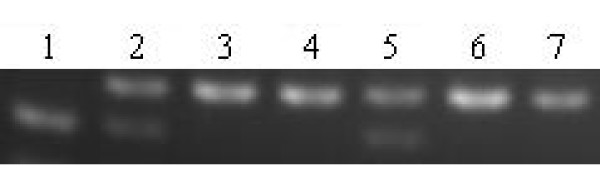
**lane 1 represents homozygous CC genotypes, lane 2 and 5 represents heterozygous AC genotypes and lane 3, 4, 6, and 7 represents homozygous AA genotypes of A20C polymorphism of AGT gene**.

**Figure 3:**
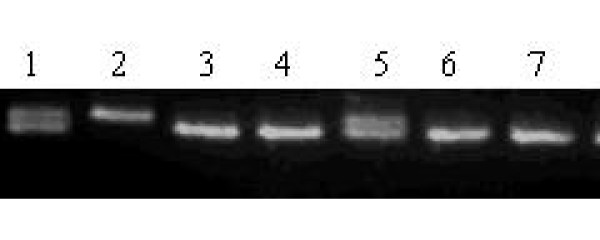
**Lane 1 and 5 represents heterozygous Gly/Trp genotypes, lane 2 represents homozygous Gly/Gly genotypes, lane 3, 4, 6 and 7 represents homozygous Trp/Trp genotypes of Gly460Trp polymorphism of alpha-adducin gene**.

**Figure 4:**
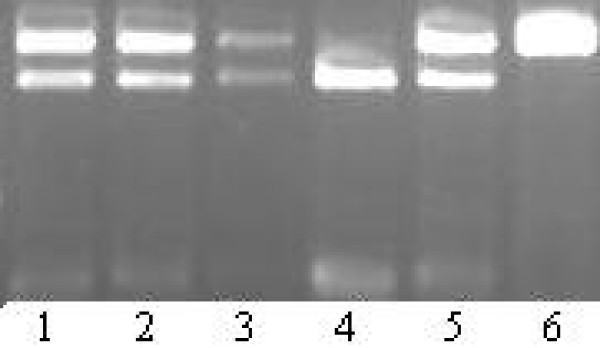
**Lane 1, 2, 3 and 5 represents heterozygous Lys/Arg genotypes, lane 4 represents homozygous Lys/Lys genotypes and lane 6 represents homozygous Arg/Arg genotypes of Lys173Arg polymorphism of aldosterone synthase gene**.

**Figure 5:**
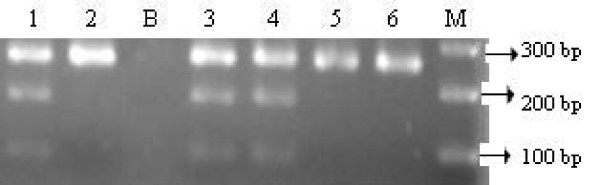
**Lane 1, 3 and 4 represents heterozygous +/- genotypes and lane 2, 5 and 6 represents homozygous -/- genotypes of MboI polymorphism of renin gene**. M represents 100 bp ladder and B represents the negative control without the template DNA.

Genetic polymorphisms involved in RAAS genes susceptibility to EHT and T2DM have been intensively scrutinized by linkage and association studies in various populations. The genes coding for each protein involved in the RAAS have been identified and studied for any relation with hypertension and other cardiovascular disorders [3,5]. Among the candidate genes of RAAS, AGT gene was considered as an important in the pathogenesis of EHT, since angiotensin II, a potent vasoconstrictor and a stimulator for aldosterone release which is obtained from the precursor molecule angiotensinogen by the proteolysis activity of renin and angiotensin converting enzyme [40]. Table 3 shows the genotypic and allelic frequency of RAAS gene polymorphisms in the subjects along with odds ratio and 95% confidence interval. There was significant association found only in A6G variant of AGT gene in Malaysian EHT with or without T2DM subjects. Figures 1, 2, 3, 4, 5 shows the genotypes of RAAS gene polymorphisms separated in gel electrophoresis.

**Table 3:** Genotypic and allelic distributions of RAAS gene polymorphisms

**Genotypes and Alleles**	**Group 1** **n (%)**	**Group 2** **n (%)**	**Group 3** **n (%)**	**Group 4** **n (%)**
A6G Genotypes AA	43 (61.43)	50 (83.30)	40 (61.54)	68 (90.67)
AG	20 (28.57)	7 (11.70)	17 (26.15)	6 (8.00)
GG	7 (10.00)	3 (5.00)	8 (12.31)	1 (1.33)
A6G Alleles C	106 (75.71)	107 (89.17)	97 (74.62)	142 (94.67)
T	34 (24.29)	13 (10.83)	33 (25.38)	8 (5.33)

p value Odds ratio (95% CI)	< 0.001* 1.76 (0.08–0.39)	0.112 0.46 (0.19–1.16)	< 0.001* 1.66 (0.07–0.37)	-

A20C Genotypes AA	46 (65.70)	37 (61.7)	45 (69.23)	53 (70.67)
AC	21 (30.00)	22 (36.7)	19 (29.23)	21 (28.00)
CC	3 (4.30)	1 (1.6)	1 (1.54)	1 (1.33)
A20C Alleles A	113 (80.70)	96 (80.00)	109 (83.80)	127 (84.67)
C	27 (19.30)	24 (20.00)	21 (16.20)	23 (15.33)

p value Odds ratio (95% CI)	0.437 0.76 (0.41–1.40)	0.336 0.72 (0.39–1.36)	0.871 0.94 (0.49–1.71)	-

*MboI *Genotypes (-/-)	46 (65.71)	43 (71.67)	47 (72.31)	56 (74.67)
(-/+)	22 (31.43)	16 (26.67)	15 (23.08)	18 (24.00)
(+/+)	2 (2.86)	1 (1.67)	3 (4.62)	1 (1.33)
*MboI *Alleles (-)	114 (81.43)	102 (85.00)	109 (83.85)	130 (86.67)
(+)	26 (18.57)	18 (15.00)	21 (16.15)	20 (13.33)

p value Odds ratio (95% CI)	0.261 0.67 (0.36–1.27)	0.727 0.87 (0.44–1.73)	0.612 0.80 (0.41–1.55)	

Lys173Arg Genotypes Lys^173^/Lys	37 (52.86)	22 (36.66)	34 (52.31)	37 (49.33)
Lys^173^/Arg	31 (44.28)	37 (61.67)	30 (46.15)	37 (49.33)
Arg^173^/Arg	2 (2.86)	1 (1.67)	1 (1.54)	1 (1.34)
Lys173Arg Alleles Lys^173^	105 (75.00)	81 (67.50)	98 (75.38)	111 (74.00)
Arg^173^	35 (25.00)	39 (32.50)	32 (24.62)	39 (26.00)

p value Odds ratio (95% CI)	0.893 1.05 (0.62–1.79)	0.280 0.73 (0.43–1.24)	0.891 1.08 (0.63–1.85)	-

Gly460Trp Genotypes Gly^460^/Gly	36 (51.43)	28 (46.67)	26 (40.00)	40 (53.33)
Gly^460^/Trp	13 (18.57)	21 (35.00)	17 (26.15)	21 (28.00)
Trp^460^/Trp	21 (30.00)	11 (18.33)	22 (33.85)	14 (18.67)
Gly460Trp Alleles Gly^460^	85 (60.71)	77 (64.17)	69 (53.08)	96 (64.00)
Trp^460^	55 (39.29)	43 (35.83)	61 (46.92)	54 (36.00)

p value Odds ratio (95% CI)	0.564 0.87 (0.54–1.40)	1.000 1.01 (0.61–1.66)	0.064 0.63 (0.39–1.03)	-

Data are reported as number of subjects with percent in parentheses, Group 1- EHT, Group 2 -T2DM, Group 3- EHT+T2DM and Group 4-Control subjects,*P value < 0.001 *vs*. Group 4.

G allele of A6G variant shows nearly 90.91% among the case subjects as compared to 9.09% of control subjects. The frequency was higher than those reported in whites but similar to that found in Japanese populations, suggesting that the A26G variant shows a significant ethnic difference [41,28]. Moreover, A6G variant found significantly associated with group 3 subjects when compared with control subjects (*P*-0.003) and was well accordance with Chilean Hispanic hypertensive subjects [42]. However, the results were also in contrast to few studies done in BMI subjects in Spanish population [43] and essential hypertensive subjects in Han, Tibetan and Yi populations [44] (p > 0.05). A previous report [33] suggested that M235T variant of AGT gene was well associated with Malaysian essential hypertensive patients. It has been well supported with the results of this study which shows that, AGT gene variants plays an important role in the pathogenesis of EHT. Tests of promoter activity and DNA interactions with factors involved in transcription initiation influence the basal rate of transcription of the gene [41]. These observations provide some biological insight about the possible mechanism by which individual differences in the AGT gene may predispose to EHT.

There was no significant association in relation to genotype or allele distributions of A20C variant of AGT gene found between the patients and the control subjects. CC genotype of A20C variant shows very less percentage (2.05%) among the case subjects as compared to 1.33% of control subjects. The results of this study were well in accordance with the previous studies done in EHT patients from a Tibetan population [8] Japanese population [10].

Based on linkage and sib-pair linkage analysis and association studies, the effects of genetic variations in the *REN *gene have been studied extensively in human population. The results of various sib-pair analyses have been unsuccessful in clearly implicating *REN *gene variations as the etiological basis of hypertension [45]. *MboI *RFLP [30] is located in intron 9 and is probably not a functional genetic variation but shown strongly positive association with hypertension in different population groups. However, a study [46] shows that genetic variations in linkage disequilibrium with this site or in a nearby gene in linkage disequilibrium may be directly implicated in an individual's genetic susceptibility to blood pressure dysregulation. In support to that, another study shows that *MboI *polymorphism was susceptibility to EHT in UAE population [30]. In contrast to those studies, our study shows that the genotypes and alleles of *MboI *polymorphism of REN gene was not statistically significant difference between the cases and control subjects (p > 0.05). The *MboI *(+) allele frequency in Malaysian hypertensive (18.57%) and normotensive (13.33%) subjects was lower than that in UAE population 51% and 36%, respectively, 20% and 24% observed in normotensive subjects in North American and Japanese population respectively. The findings of *MboI *polymorphism of *REN *gene of this study was similar to the few studies which failed to show an association between hypertensive and normotensive subjects [47].

Several studies supported that, the variant in adducin gene in rats affects renal function by modulating the overall capacity of tubular epithelial cells to transport ions modifying the assembly of the actin cytoskeleton [48]. Few studies revealed that, the possible association of Trp allele of Gly460Trp polymorphism of ADD1 was associated with an increased risk of EHT and T2DM [20]. In the present study, we could not detect any association of Trp allele of Gly460Trp polymorphism of ADD1 in Malaysian EHT with or without T2DM subjects. No association was found between the Gly460Trp allele and hypertension in Japanese population [29].

In relation with T2DM the genotypes and alleles of Gly460Trp polymorphism shows no significant difference with control subjects (*P *= 1.000) which is similar to study done in Irish population [49] in relation with diabetic nephropathy and Type 1 diabetes (*P *= 0.087). However, there are still remains the possibility of ADD1 gene affects membrane ion transport because we did not investigate the effect on salt-sensitivity or response to diuretic treatment. However, several studies suggest that a variant in ADD1 gene on its own is insufficient to develop hypertension [19,20].

The three common polymorphic variants of CYP11B2; C-344T at 5' distal promoter region of the gene, a mutation in intron 2, and K173R (Lys173Arg) in exon 3 have been extensively evaluated in relation to hypertension and cardiovascular disease. Unpublished data shows that, C344T variant of CYP11B2 is not associated with hypertension in Malays; an ethnic population in Malaysia. Hence, this study focus on the other variant Lys173Arg of CYP11B2 gene located at exon 3 which has been associated with low renin hypertensive patients in Chilean populations [42]. In this study, the frequency of the variant A allele of Lys173Arg polymorphism was seen very less in hypertensive patients with T2DM (24.62%) as compared to control subjects (26.00%). No difference in AA genotype frequency was evident between hypertensive patients (2.86%) and control subjects (1.34%). Our results were totally diverged from the previous report [42] shows that Arg 173 allele frequency (21%) in low renin hypertensive Chilean subjects.

Table 4 shows the genotypic and allelic distributions of RAAS gene polymorphisms between male and female subjects. There was no significant difference was found in either genotypic or allelic distribution in both male and female subjects (p > 0.05).

**Table 4:** Allelic distribution of RAAS gene polymorphisms in gender

		**Group 1**		**Group 2**		**Group 3**		**Group 4**	
**Alleles of RAAS Gene Polymorphisms**		**Male**	**Female**	**Male**	**Female**	**Male**	**Female**	**Male**	**Female**
A6G	C	76 (78.40)	26 (21.60)	60 (90.90)	6 (9.10)	61 (76.25)	19 (23.75)	74 (94.87)	4 (5.13)
	T	30 (89.50)	8 (10.50)	47 (87.04)	7 (12.96)	36 (65.45)	14 (25.45)	68 (94.44)	4 5.56)

p value OR (95% CI)		0.662 0.78 (0.318–1.914)		0.563 1.49 (0.469–4.729)		0.680 1.25 (0.559–2.790)		1.000 1.09 (0.262–4.522)	

A20C	A	82 (80.40)	20 (19.60)	55 (83.30)	11 (16.70)	70 (87.50)	10 (12.50)	67 (85.90)	11 (14.10)
	C	31 (81.60)	7 (18.40)	41 (76.00)	13 (24.00)	39 (78.00)	11 (22.00)	60 (83.33)	12 (1.67)

p value OR (95% CI)		1.000 0.93 (0.356–2.045)		0.363 1.59 (0.645–3.896)		0.220 1.97 (0.770–5.063)		0.821 1.22 (0.501–2.964)	

*MboI*	(-)	85 (83.33)	17 (16.67)	58 (87.88)	8 (12.12)	66 (82.50)	14 (17.50)	65 (83.33)	13 (16.67)
	(+)	29 (76.32)	9 (23.68)	44 (81.48)	10 (18.52)	43 (86.00)	7 (14.00)	65 (90.28)	7 (9.72)

p value OR (95% CI)		0.339 1.55 (0.624–3.860)		0.442 1.65 (0.601–4.519)		0.635 0.77 (0.287–2.056)		0.238 0.54 (0.202–1.436)	

Lys173Arg	Lys^173^	76 (74.51)	26 (25.49)	44 (66.70)	22 (33.30)	59 (73.75)	21 (26.25)	58 (74.36)	20 (25.64)
	Arg^173^	29 (76.30)	9 (23.70)	37 (68.52)	17 (31.48)	39 (78.00)	11 (22.00)	53 (73.61)	19 (26.39)

p value OR (95% CI)		1.000 0.90 (0.380–2.166)		0.847 0.92 (0.426–1.983)		0.678 0.79 (0.344–1.825)		1.000 1.04 (0.501–2.157)	

Gly460Trp	Gly^460^	66 (64.71)	36 (35.29)	42 (63.64)	24 (36.36)	46 (57.50)	34 (42.50)	52 (69.30)	26 (30.70)
	Trp^460^	19 (50.00)	19 (50.00)	35 (64.81)	19 (35.19)	23 (46.00)	27 (54.00)	44 (61.11)	28 (38.89)

p value OR (95% CI)		0.124 1.88 (0.862–3.899)		0.893 0.95 (0.458–2.012)		0.201 1.59 (0.780–3.234)		0.479 1.27 (0.653–2.482)	

Data are reported as number of subjects with percent in parentheses, OR-odds ratio, CI – Confidence interval, Group 1- EHT, Group 2 -T2DM, Group 3- EHT+T2DM and Group 4-Control subjects, P value > 0.05 *vs*. Group 4.

Some evidence shows that modulation of the RAAS by use of angiotensin-converting enzyme inhibitors or angiotensn II receptor blockers leads to improved insulin sensitivity, glycemic control and possibly prevention of T2DM [50]. This study failed to show significant association between all the five genetic polymorphisms of RAAS genes with T2DM. The reason might be the different methods used or may occur due to racial differences or heterogeneity of the population sampling bias or possibly due to the environmental factors may contribute to the negative associations [51]. Moreover, despite the considerable controversy regarding the existence and importance of ethnic differences in genetic effects for complex diseases, it seems evident that genetic markers for proposed gene-disease associations vary in frequency across populations. Association studies must be repeated in this population to ensure that any association of candidate genes with T2DM is pathogenetically significant.

### Study limitations

The present study has to be interpreted within the context of its limitations. The present study provided only the evidences of the association between the genetic polymorphisms of RAAS genes with EHT and T2DM at the gene level and we did not address the mechanism or the functionality of the variants. The control subjects are not age, sex matched and they are relatively young as compared to case subjects. Further association/replication studies with well-designed larger sample size subjects involving other polymorphisms of RAAS genes in related to cardiovascular diseases is recommended.

## Conclusion

Among the five polymorphisms of RAAS genes, only A6G variant of AGT gene was significantly associated in EHT with or without T2DM, other polymorphisms were failed to show any significant association between the case and control subjects. In conclusion, the present study implies that genotyping for the A6G variant of AGT gene could in the future become an important part of the clinical process of risk identification for EHT and T2DM in Malaysian population.

## Abbreviations

AGT: Angiotensinogen; BMI: Body mass index; CAD: Coronary artery disease; EHT: Essential hypertension; DBP: Diastolic Blood Pressure; FPG: Fasting plasma glucose level; HDL-C: High density lipoprotein cholesterol; K: Potassium; LDL-C: Low density lipoprotein cholesterol; Na: Sodium; PCR: Polymerase chain reaction; RAAS: Renin Angiotensin Aldosterone System; *REN: *Renin; RFLP: Restriction fragment length polymorphism; T2DM: Type 2 Diabetes Mellitus; SBP: Systolic blood pressure; TG: Triglycerides and TC: Total cholesterol.

## Competing interests

The authors declare that they have no competing interests.

## Authors' contributions

RV and PI conceived the study and RV participated in the experimental design, data acquisition and analysis, interpretation of results, and drafted the manuscript. PI, JS and NS interpreted the results and critically reviewed the study for important intellectual content. All authors approved the final version of the manuscript.
